# Long-Term Outcomes after Switching to Tenofovir Alafenamide in Patients with Chronic Hepatitis B

**DOI:** 10.3390/ijms25042245

**Published:** 2024-02-13

**Authors:** Tomohiro Nishikawa, Masahiro Matsui, Saori Onishi, Kosuke Ushiro, Akira Asai, Soo-Ki Kim, Hiroki Nishikawa

**Affiliations:** 1 Second Department of Internal Medicine, Osaka Medical and Pharmaceutical University, Takatsuki 569-8686, Japankawakaminmin19910829@yahoo.co.jp (S.O.);; 2 Department of Gastroenterology, Kobe Asahi Hospital, Kobe 653-8501, Japan; kinggold@kobe-asahi-hp.com

**Keywords:** chronic hepatitis B, tenofovir alafenamide, switch, efficacy, safety

## Abstract

We sought to determine the long-term outcomes of chronic hepatitis B (CHB) cases switching to tenofovir alafenamide (TAF, *n* = 104, median age = 63.5 years). Data at switching to TAF (baseline) and those at 1, 2, 3, 4, and 5 years from switching to TAF were compared. At baseline, HB envelop antigen (HBeAg) seropositivity was found in 20 patients (19.2%), and undetectable HBV-DNA in 77 patients (74.0%). Percentage of detectable HBV-DNA significantly reduced at any time point. HB surface antigen (HBsAg) levels significantly reduced at 3, 4, and 5 years. The percentage of HBeAg seropositivity significantly reduced at 5 years. HB core related antigen levels did not significantly change. In patients with baseline HbeAg seropositivity, HbsAg levels significantly reduced at any time point, and a similar trend was found in patients without HBeAg seropositivity. In patients with baseline FIB4 index >1.85, HBsAg levels significantly reduced at 3, 4, and 5 years, and in patients with baseline FIB4 index <1.85, HBsAg levels significantly reduced at any time point. The estimated glomerular filtration rate significantly reduced only at 5 years. The discontinuation rate owing to the side effects of TAF was 0%. In conclusion, switching to TAF therapy in patients with CHB may be effective and safe at least up to 5 years.

## 1. Introduction

Hepatitis B virus (HBV) is a highly infectious virus, with an estimated 1 in 3.5 people worldwide infected or previously infected [1]. The therapeutic goals of antiviral therapy for patients with persistent HBV infection are to avoid chronic liver failure and to prevent the development of hepatocellular carcinoma by suppressing hepatitis activity and the development of liver fibrosis, thereby improving life expectancy and quality of life [2,3]. Five nucleoside analogues (NAs) against HBV are currently approved in Japan: adefovir (ADV), entecavir (ETV), tenofovir disoproximal fumarate (TDF), and tenofovir alafenamide (TAF), starting with lamivudine (LAM), approved in 2000 [4]. Like TDF, TAF is a prodrug of tenofovir, but its selective uptake by the liver has been shown to produce similar effects to those of TDF at doses less than one-tenth those of TDF, reducing the risk of renal dysfunction and loss of bone density [5,6,7,8]. Because of these advantages, TAF is currently used as a first-line drug in the treatment of chronic hepatitis B (CHB), along with ETV.

More than six years have passed since TAF treatment for patients with CHB was covered by insurance in Japan [8]. Due to the superior efficacy and safety of TAF, the number of CHB cases switching to TAF has been increasing in recent years [9,10,11,12,13,14]. In a comparative study by Li et al. between the two groups of patients who switched from ETV to TAF and those who continued ETV, they found that the rate of alanine aminotransferase (ALT) normalization after one year was better in patients who switched to TAF than in those who continued ETV, and there were no tolerability problems [15]. Ogawa et al. reported that patients who switched from Nas, such as ETV and TDF, to TAF had good efficacy and safety up to three years after the switch [16]. The attenuation of HB surface antigen (HBsAg) levels is also said to be better with TAF than with ETV [17]. In principle, ETV is taken orally on an empty stomach, but TAF can exert its effects regardless of food intake [17]. Current guidelines recommend switching to TAF as one of the recommended treatments due to the emergence of resistant strains or safety concerns, even if the treatment response is favorable with ETV, LAM, TDF, or LAM plus ADV combination therapy [18].

However, data for long-term outcomes of TAF treatment beyond 3 years after switching to TAF in patients with CHB are currently limited [14]. The purpose of this study is to determine the long-term outcomes of CHB cases who switched to TAF.

## 2. Results

Baseline characteristics at switching to TAF (55 men (52.9%)) in this study are demonstrated in Table 1. Switching from ETV was observed in 71 cases, from TDF in 28 cases, from LAM in 2 cases, from LAM plus ADV combination therapy in 2 cases, and from pegylated interferon in 1 case. Cirrhosis was found in 27 patients (26.0%). HB envelop antigen (HBeAg) seropositivity was found in 20 patients (19.2%). With regard to baseline HBV-DNA level at switching to TAF, undetectable HBV-DNA level was found in 77 patients (74.0%). In patients with detectable HBV-DNA level at switching to TAF (n = 27), the median HBV-DNA level was 1.3 log IU/mL. With regard to HB core related antigen (HBcrAg) level at switching to TAF (missing data, n = 21), <3.0 log U/mL, ≥3.0 and <4.0 log U/mL, ≥4.0 and <5.0 log U/mL, and ≥5.0 log U/mL was found in 41 (49.4%), 13 (15.7%), 13 (15.7%), and 16 patients (19.3%), respectively. The median time from the time of initial treatment for HBV to the switch to TAF was 5.4 years. The median follow-up period from switching to TAF to last follow-up date was 4.8 years. During the observation period, no TAF-related serious adverse events were observed. The discontinuation rate owing to the side effects of TAF was thus 0%. During the observation period, loss of HB surface antigen was found in 4 patients (3.8%).

### 2.1. Changes in ALT Levels over Time

**Figure 1:**
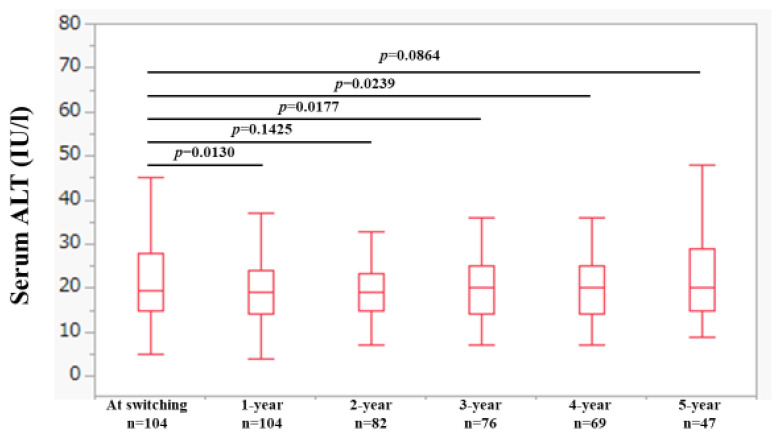
Changes in ALT levels over time.

Changes in ALT levels over time are shown in Figure 1. The median interquartile range (IQR) ALT levels at baseline (n = 104), 1 year (n = 104), 2 years (n = 82), 3 years (n = 76), 4 years (n = 69) and 5 years (n = 47) were 19.5 (15, 27.8) IU/L, 19 (14, 24) IU/L, 19 (15, 23.3) IU/L, 19 (15, 23) IU/L, 20 (14, 25) IU/L, and 20 (15, 29) IU/L, respectively, (*p* values (vs. baseline): *p* = 0.0130 (1 year), *p* = 0.1425 (2 years), *p* = 0.0177 (3 years), *p* = 0.0239 (4 years), and *p* = 0.0864 (5 years).

### 2.2. Changes in FIB4 Index over Time

**Figure 2:**
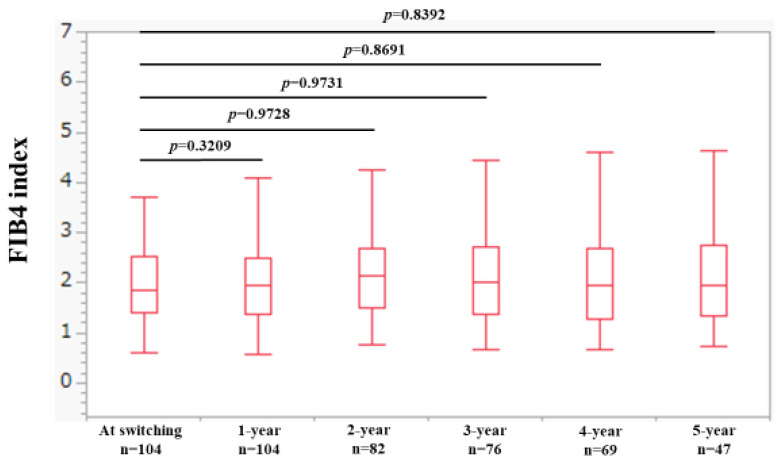
Changes in FIB4 index over time.

Changes in FIB4 index over time are shown in Figure 2. The median (IQR) FIB4 index at baseline (n = 104), 1 year (n = 104), 2 years (n = 82), 3 years (n = 76), 4 years (n = 69), and 5 years (n = 47) were 1.85 (1.41, 2.53), 1.95 (1.36, 2.50), 2.15 (1.51, 2.69), 2.01 (1.36, 2.73), 1.93 (1.27, 2.70), and 1.95 (1.35, 2.75), respectively, (*p* values (vs. baseline): *p* = 0.3209 (1 year), *p* = 0.9728 (2 years), *p* = 0.9731 (3 years), *p* = 0.8691 (4 years), and *p* = 0.8392 (5 years).

### 2.3. Changes in eGFR Levels over Time

**Figure 3:**
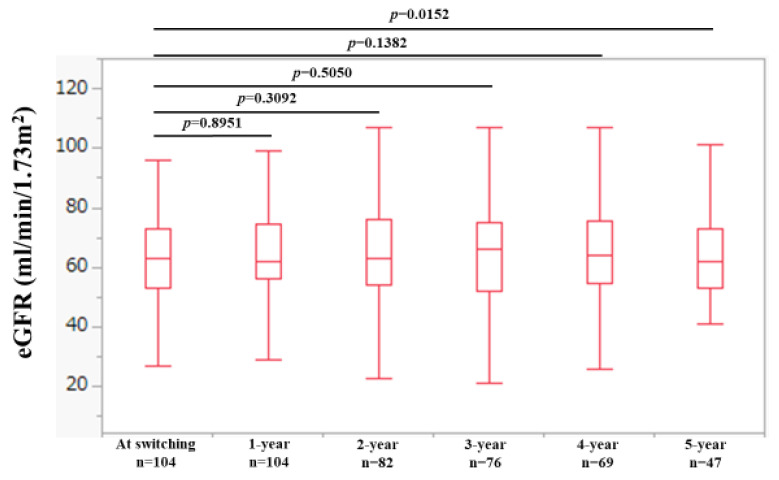
Changes in eGFR levels over time.

Changes in estimated glomerular filtration rate (eGFR) over time are shown in Figure 3. The median (IQR) eGFR at baseline (n = 104), 1 year (n = 104), 2 years (n = 82), 3 years (n = 76), 4 years (n = 69), and 5 years (n = 47) were 63 (53.3, 72.8) mL/min/1.73 m^2^, 62 (56, 74.8) mL/min/1.73 m^2^, 63 (54, 76.3) mL/min/1.73 m^2^, 66 (52.3, 75) mL/min/1.73 m^2^, 64 (54.5, 75.5) mL/min/1.73 m^2^, and 62 (63, 73) mL/min/1.73 m^2^, respectively, (*p* values (vs. baseline): *p* = 0.8951 (1 year), *p* = 0.3092 (2 years), *p* = 0.5050 (3 years), *p* = 0.1382 (4 years), and *p* = 0.0152 (5 years).

### 2.4. Changes in HBsAg Levels over Time

**Figure 4:**
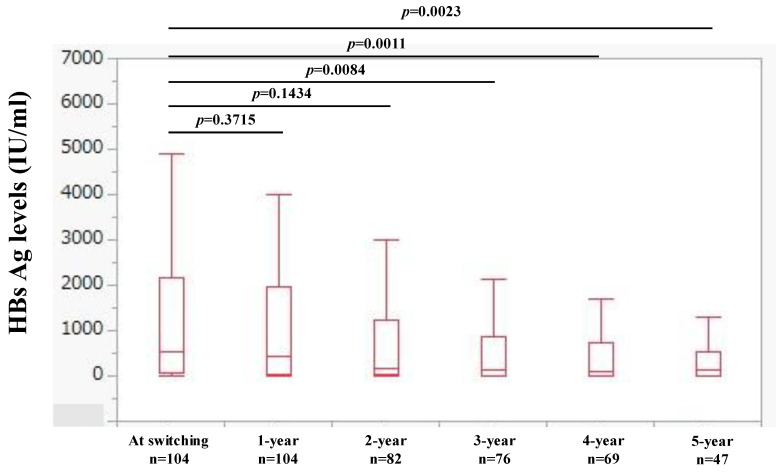
Changes in HBsAg levels over time.

Changes in HBsAg levels over time are shown in Figure 4. The median (IQR) HBsAg levels at baseline (n = 104), 1 year (n = 104), 2 years (n = 82), 3 years (n = 76), 4 years (n = 69), and 5 years (n = 47) were 549.6 (50.2, 2159.7) IU/mL, 440.1 (27.6, 1960.0) IU/mL, 175.6 (22.5, 1231.7) IU/mL, 143 (10.7, 859.7) IU/mL, 107 (2.4, 730.5) IU/mL, and 128 (3.0, 530) IU/mL, respectively, (*p* values (vs. baseline): *p* = 0.3715 (1 year), *p* = 0.1434 (2 year), *p* = 0.0084 (3 years), *p* = 0.0011 (4 years), and *p* = 0.0023 (5 years).

### 2.5. Changes in the Percentage of Detectable HBV-DNA over Time

**Figure 5:**
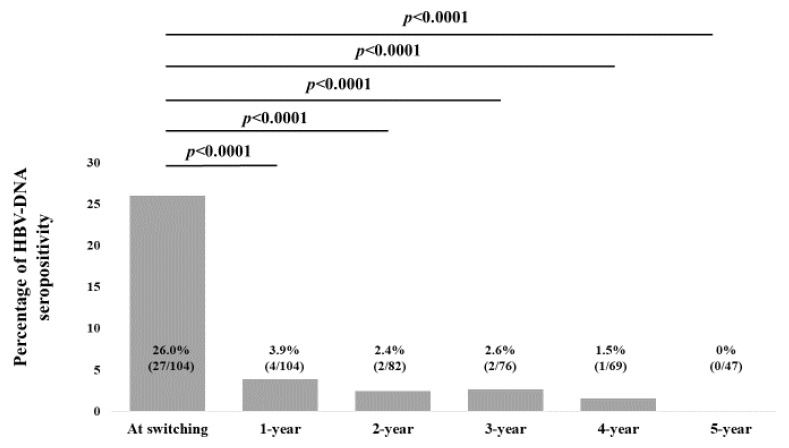
Changes in the percentage of detectable HBV-DNA over time.

Changes in the percentage of detectable HBV-DNA over time are shown in Figure 5. The percentage of detectable HBV-DNA at baseline (n = 104), 1 year (n = 104), 2 years (n = 82), 3 years (n = 76), 4 years (n = 69), and 5 years (n = 47) were 26.0% (27/104), 3.9% (4/104), 2.4% (2/82), 2.6% (2/76), 1.5% (1/69), and 0% (0/47), respectively, (*p* values (vs. baseline): *p* < 0.0001 (1 year), *p* < 0.0001 (2 years), *p* < 0.0001 (3 years), *p* < 0.0001 (4 years), and *P* < 0.0001 (5 years).

### 2.6. Changes in the Percentage of HBeAg Seropositivity over Time

**Figure 6:**
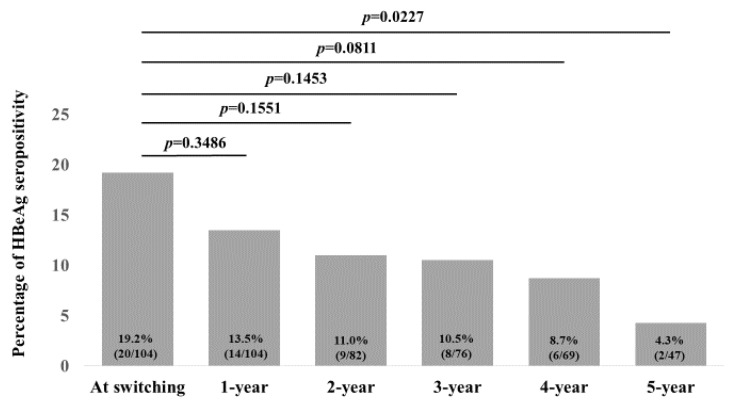
Changes in the percentage of HBeAg seropositivity over time.

Changes in the percentage of HBeAg seropositivity over time are shown in Figure 6. The percentage of HBeAg seropositivity at baseline (n = 104), 1 year (n = 104), 2 years (n = 82), 3 years (n = 76), 4 years (n = 69), and 5 years (n = 47) were 19.2% (20/104), 13.5% (14/104), 11.0% (9/82), 10.5% (8/76), 8.7% (6/69), and 4.3% (2/47), respectively, (*p* values (vs. baseline): *p* = 0.3486 (1 year), *p* = 0.1551 (2 years), *p* = 0.1453 (3 years), *p* = 0.0811 (4 years), and *p* = 0.0227 (5 years).

### 2.7. Changes in the Distribution of HBcrAg Levels over Time

**Figure 7:**
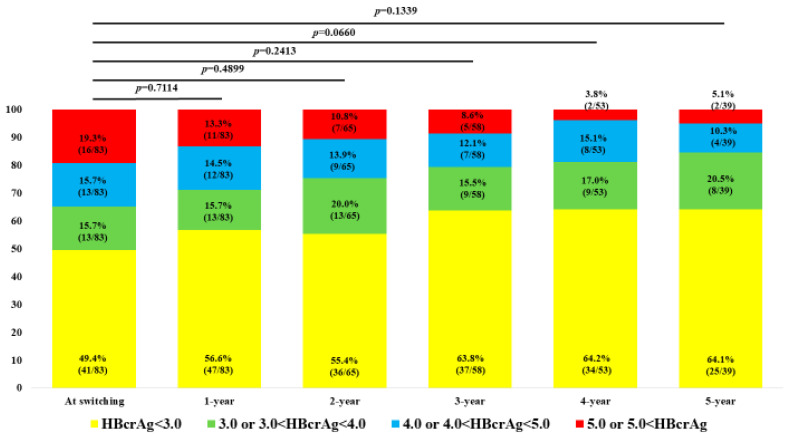
Changes in the distribution of HBcrAg levels over time. Pearson’s χ-square test was used for between-group comparisons.

Changes in the distribution of HBcrAg levels over time are shown in Figure 7. At 1 year (n = 83), <3.0 log U/mL, ≥3.0 and <4.0 log U/mL, ≥4.0 and <5.0 log U/mL. and ≥5.0 log U/mL was found in 47 (56.7%), 13 (15.7%), 12 (14.5%), and 11 patients (13.3%), respectively. At 2 years (n = 65), <3.0 log U/mL, ≥3.0 and <4.0 log U/mL, ≥4.0 and <5.0 log U/mL. and ≥5.0 log U/mL was found in 36 (55.4%), 13 (20.0%), 9 (13.9%), and 7 patients (10.8%), respectively. At 3 years (n = 58), <3.0 log U/mL, ≥3.0 and <4.0 log U/mL, ≥4.0 and <5.0 log U/mL. and ≥5.0 log U/mL was found in 37 (63.8%), 9 (15.5%), 7 (12.1%), and 5 patients (8.6%), respectively. At 4 years (n = 53), <3.0 log U/mL, ≥3.0 and <4.0 log U/mL, ≥4.0 log and <5.0 log U/mL. and ≥5.0 log U/mL was found in 34 (64.2%), 9 (17.0%), 8 (15.1%), and 2 patients (3.8%), respectively. At 5 years (n = 39), <3.0 log U/mL, ≥3.0 log and <4.0 log U/mL, ≥4.0 and <5.0 log U/mL. and ≥5.0 log U/mL was found in 25 (64.1%), 8 (20.5%), 4 (10.3%), and 2 patients (5.1%), respectively. No significant differences were found when comparing baseline to any time period.

### 2.8. Changes in HBsAg Levels over Time in Patients with and without HBeAg Seropositivity at Switching to TAF

**Figure 8:**
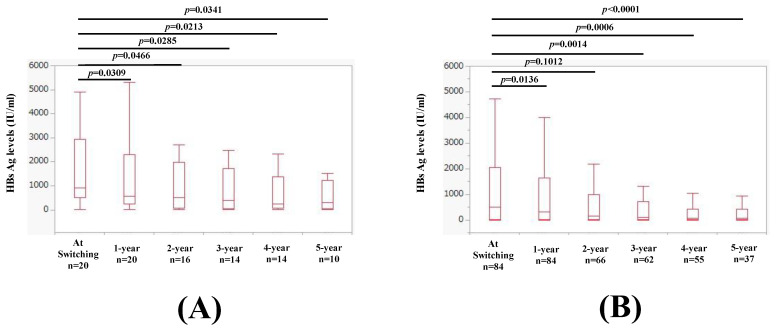
(**A**) Changes in HBsAg levels over time in patients with baseline HBeAg seropositivity at switching to TAF. (**B**) Changes in HBsAg levels over time in patients without baseline HBeAg seropositivity at switching to TAF.

Changes in HBsAg levels over time in patients with HBeAg seropositivity at switching to TAF are shown in Figure 8A. The median (IQR) HBsAg levels at baseline (n = 20), 1 year (n = 20), 2 years (n = 16), 3 years (n = 14), 4 years (n = 14), and 5 years (n = 10) were 905.5 (503.8, 2949.8) IU/mL, 574.9 (242.6, 2292.0) IU/mL, 500.5 (86.8, 1981.8) IU/mL, 399.1 (48, 1709.7) IU/mL, 243.5 (85.5, 1371.6) IU/mL, and 293 (37.1, 1235) IU/mL, respectively, (*p* values (vs. baseline): *p* = 0.0309 (1 year), *p* = 0.0466 (2 years), *p* = 0.0285 (3 years), *p* = 0.0213 (4 years), and *p* = 0.0341 (5 years).

Changes in HBsAg levels over time in patients without HBeAg seropositivity at switching to TAF are shown in Figure 8B. The median (IQR) HBsAg levels at baseline (n = 84), 1 year (n = 84), 2 years (n = 66), 3 years (n = 62), 4 years (n = 55), and 5 years (n = 37) were 495.4 (26.9, 2042.4) IU/mL, 317.8 (9.6, 1642.8) IU/mL, 143.5 (13.1, 999.1) IU/mL, 96.5 (6.3, 723) IU/mL, 74 (1.4, 426) IU/mL, and 66.9 (1.3, 414.1) IU/mL, respectively, (*p* values (vs. baseline): *p* = 0.0136 (1 year), *p* = 0.1012 (2 years), *p* = 0.0014 (3 years), *p* = 0.0006 (4 years), and *p* < 0.0001 (5 years).

### 2.9. Changes in HBsAg Levels over Time in Patients with FIB4 Index >1.85 and <1.85 at Switching to TAF

**Figure 9:**
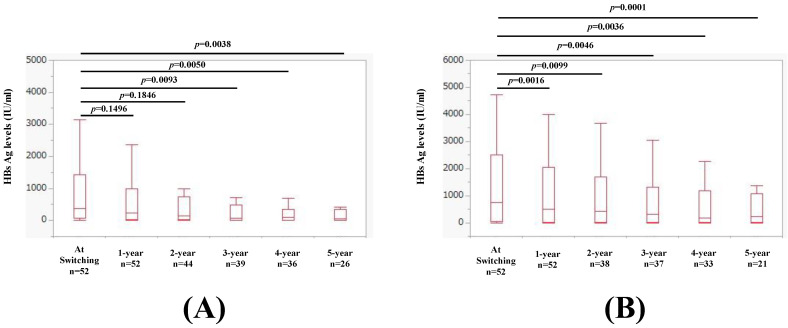
(**A**) Changes in HBsAg levels over time in patients with baseline FIB4 index >1.85 at switching to TAF. (**B**) Changes in HBsAg levels over time in patients with baseline FIB4 index <1.85 at switching to TAF.

The median FIB4 index at the switch to TAF was 1.85. Changes in HBsAg levels over time in patients with FIB4 index >1.85 at switching to TAF are shown in Figure 9A. The median (IQR) HBsAg levels at baseline (n = 52), 1 year (n = 52), 2 years (n = 44), 3 years (n = 39), 4 years (n = 36), and 5 years (n = 26) were 376.2 (62.9, 1424) IU/mL, 222.3 (27.6, 999.1) IU/mL, 133 (25.4, 740) IU/mL, 82 (7, 477) IU/mL, 84.5 (1.5, 357.8) IU/mL, and 52.5 (0.9, 356.8) IU/mL, respectively, (*p* values (vs. baseline): *p* = 0.1496 (1 year), *p* = 0.1846 (2 years), *p* = 0.0093 (3 years), *p* = 0.0050 (4 years), and *p* = 0.0038 (5 years).

Changes in HBsAg levels over time in patients with FIB4 index <1.85 at switching to TAF are shown in Figure 9B. The median (IQR) HBsAg levels at baseline (n = 52), 1 year (n = 52), 2 years (n = 38), 3 years (n = 37), 4 years (n = 33), and 5 years (n = 21) were 749.2 (40, 2513.5) IU/mL, 498.6 (25.8, 2045.7) IU/mL, 434 (18.8, 1681.8) IU/mL, 310 (13.9, 1308.1) IU/mL, 187 (15.8, 1171.7) IU/mL, and 243 (22.1, 1068.5) IU/mL, respectively, (*p* values (vs. baseline): *p* = 0.0016 (1 year), *p* = 0.0099 (2 years), *p* = 0.0046 (3 years), *p* = 0.0036 (4 years), and *p* = 0.0001 (5 years).

## 3. Discussion

Summary of the results regarding the efficacy and safety of TAF therapy in this study are as follows: (1) ALT remained significantly lower in the 1-, 3-, and 4-year follow-up cases (n = 76, 69 and 47) than that at the time of switching, (2) no significant change in FIB4 index was observed over time during the follow-up period, (3) HBsAg levels decreased significantly in the 3-, 4-, and 5-year follow-up cases compared to those at the time of the switching, and a similar trend (i.e., significant decrease in HBsAg levels) was observed by HBeAg seropositivity or FIB4 index, (4) the percentage of HBV-DNA positivity significantly decreased over time during the follow-up period, (5) the percentage of HBeAg seropositivity gradually decreased, and at 5 years, significant reduction was found, (6) the proportion of cases with HBcrAg <3.0 did not change much at any time point, (7) although eGFR was significantly lower in the 5-year follow-up cases than that at the time of switching, there were no cases of discontinuation due to TAF-related side effects. Ogawa et al. reported that patients who switched from ETV or TDF to TAF had good efficacy and safety up to three years after the switch [16]. Based on the results of our study, the efficacy (especially in HBV-related markers other than HBcrAg) and safety of TAF treatment should be maintained for at least up to five years after the switch to TAF. This is the highlight of our study. In the recent Chinese report by Chan et al., long-term TAF therapy (up to five years after the switch to TAF) resulted in high rates of HBV suppression, no remarkable resistance, and favorable safety, which are in line with our current results [14]. HBsAg reduction or HBsAg loss is related to functional cure of CHB [19,20]. In our study, we also examined HBcr antigen trends over time, and this is the major difference between these two studies.

On the other hand, Ogawa et al. reported that eGFR improved in patients switched to TAF, but no such trend was observed in this study [16]. Differences in background factors between these two studies may be related to the differences in the improvement of eGFR after the switch to TAF. Cirrhotic cases accounted for 7.9% in the study by Ogawa et al. vs. 26.0% (27/104) in the present study. The median age was 59 years in the Ogawa et al. study vs. 63.5 years in the present study.

HBcrAg is the generic name for three antigenic component proteins: HBc antigen translated from pregenomic mRNA, HBeAg translated from precore mRNA, and p22cr antigen [21]. HBcrAg, like HBsAg levels, is also a predictive marker for carcinogenesis and is of clinical importance [22,23,24]. HBcrAg also correlates well with intrahepatic covalently closed circular DNA [25]. Despite the significant decrease in the percentage of HBeAg-positive cases after the switch in this study, there was no significant decrease in HBcrAg levels at any time point compared with those at the time of switching to TAF. The reasons for these are unclear, and further examinations will be necessary to confirm the impact of TAF therapy on the HBcrAg levels. Switching to TAF can obtain significant HBsAg reduction in HBeAg-negative patients, which is in agreement with our results [26]. Although the number of cases in this study was small (n = 20), a significant decrease in HBsAg levels was observed even in HBeAg-positive cases at any time point compared with those at the time of switching, which is very important information for clinicians.

The significant difference of age in patients with FIB4 index >1.85 and <1.85 at the time of switching may suggest that patients with advanced liver fibrosis have longer disease duration of hepatitis B [27]. Despite this, the attenuation of HBsAg levels was similar between the two groups when examined by FIB4 index. Treatment efficacy in cases of switching to TAF may not be affected by the disease duration. In this study, there were the two patients who switched from LAM plus ADV combination therapy to TAF, and they were both HbeAg-positive at the time of the switch, and after the switch to TAF, HBeAg became negative and HBV-DNA remained undetectable during the observation period. Data on switching from LAM plus ADV combination therapy to TAF in patients with CHB are limited [18]. However, considering the results of our two cases, switching from LAM plus ADV combination therapy to TAF therapy may be effective.

We must acknowledge several limitations to this study. First, this was a single-arm and single-center observational study with a retrospective analysis. Second, the study included 104 CHB cases of switching to TAF, but not all of them have been followed for 5 years. Third, HBcrAg level was unmeasured in some cases. Fourth, this study did not compare cases switching to TAF with those continuing TDF or ETV. Finally, various types of HBV drugs were administered prior to switching to TAF, creating biases. Thus, data should be interpreted with caution. However, the current results demonstrated that the switch to TAF for the treatment of CHB maintains efficacy and safety over the long term.

In conclusion, switching to TAF therapy in patients with CHB may be effective and safe at least up to 5 years.

## 4. Patients and Methods

### 4.1. Patients

The subjects were 104 consecutive patients with CHB switching to TAF therapy (25 mg per day) at our hospital between July 2017 and June 2022. Cirrhosis was determined by the radiological findings. The reasons for switching to TAF are as follows: (1) in patients receiving ETV, considering the method of administration (ETV is taken orally on an empty stomach; TAF has no particular restrictions) or the possibility of resistant strains emerging, (2) in patients receiving TDF, considering the effect on renal function, (3) in patients receiving LAM or LAM plus ADV combination therapy, considering the emergence of resistant strains, (4) in patients receiving pegylated interferon, considering the virological effects. During the follow-up period, HBV-related markers (HBV-DNA level, HBeAg seropositivity, HBsAg level, HBcrAg level) were monitored on a regular basis. The institutional review board of Osaka Medical and Pharmaceutical University hospital provided ethical approval (approval number, 2023-158). Since this study involved a retrospective nature, direct face-to-face informed consent to the patients with regard to this study was waived. The protocol of the current study strictly adhered to all contents of the Declaration of Helsinki in 1975.

### 4.2. Blood Testing

HBsAg levels, HBeAg levels, HBcrAg levels, and anti-HBe levels were tested by enzyme-linked immunosorbent assay (ELISA) or Chemiluminescent Enzyme Immunoassay. HBV-DNA levels were tested by COBAS TaqMan HBV Test (Roche Diagnostics K.K., Tokyo, Japan). In some cases, we used iTACT-HBcrAg test [27]. Lower detection limit of HBcrAg was <3.0 log U/mL, whereas that of iTACT HBcrAg was <2.1 log U/mL.

### 4.3. Statistics

Continuous data were presented by median value (IQR). Data at switching to TAF (baseline) and those at 1, 2, 3, 4, and 5 years from switching to TAF were compared. Paired *t*-test was used for comparisons of continuous variables. Pearson’s χ-square test was used for between-group comparisons. A *p* = 0.05 was set at the significant level by the JMP ver. 16 (SAS Institute Inc., Cary, NC, USA).

## Figures and Tables

**Table 1:** Baseline data (n = 104, at the time of switching to TAF).

Variables	Number or Median (IQR)
Gender (male/female)	55/49
Age (years)	63.5 (54, 72)
Body mass index (kg/m^2^)	23.2 (20.7, 25.8)
Alanine aminotransferase (IU/L)	19.5 (15, 27.8)
FIB4 index	1.85 (1.41, 2.53)
eGFR (mL/min/1.73 m^2^)	63 (53.3, 72.8)
Alfa-fetoprotein (ng/mL)	2.3 (1.6, 3.1)
Presence of cirrhosis, yes/no	27/77
HBsAg levels at switching to TAF (IU/mL)	549.6 (50.2, 2159.7)
HBeAg seropositivity at switching to TAF, yes/no	20/84
Positivity for HBV-DNA at switching to TAF, yes/no	27/77
HBcrAg levels at switching to TAF (missing data, n = 21)	
<3.0 Log U/mL	41
3.0≤ and <4.0 Log U/mL	13
4.0≤ and <5.0 Log U/mL	13
5.0 Log U/mL	16

TAF; tenofovir alafenamide, IQR; interquartile range, FIB4; fibrosis-4 (liver fibrosis marker), eGFR; estimated glomerular filtration rate, HBsAg; hepatitis B surface antigen, HBeAg; hepatitis B envelop antigen, HBcrAg; hepatitis B core related antigen.

## Data Availability

The data presented in this study are available on request from the corresponding author (accurately indicate status).

## Abbreviations

HBV; hepatitis B virus, NA; nucleoside analogue, ADV; adefovir, ETV; entecavir, TDF; tenofovir disoproximal fumarate, TAF; tenofovir alafenamide, LAM; lamivudine, CHB; chronic hepatitis B, ALT; alanine aminotransferase, HBeAg; HB envelop antigen, HBsAg; HB surface antigen, HBcrAg; HB core related antigen, IQR; interquartile range, eGFR; estimated glomerular filtration rate.
